# Molecular Characterization of Microbial and Fungal Communities on Dry-Aged Beef of Hanwoo Using Metagenomic Analysis

**DOI:** 10.3390/foods9111571

**Published:** 2020-10-29

**Authors:** Sangdon Ryu, Minhye Shin, Soohyun Cho, Inho Hwang, Younghoon Kim, Sangnam Oh

**Affiliations:** 1 Department of Animal Science and Institute of Milk Genomics, Jeonbuk National University, Jeonju 54896, Korea; rsangdon@jbnu.ac.kr (S.R.); inho.hwang@jbnu.ac.kr (I.H.); 2 Department of Agricultural Biotechnology and Research Institute of Agriculture and Life Science, Seoul National University, Seoul 08826, Korea; alsgp01@gmail.com; 3 Animal Products and Utilization Division, National Institute of Animal Science, Rural Development Administration, Wanju 55365, Korea; shc0915@korea.kr; 4 Department of Functional Food and Biotechnology, Jeonju University, Jeonju 55069, Korea

**Keywords:** microbiome, mycobiome, dry aging, lactic acid bacteria

## Abstract

Dry aging has been widely applied for the aging of meat to produce a unique flavor and tenderness of meat. A number of microorganisms are present, forming a community with interactions that affect the meat aging process. However, their comprehensive compositions are still not well understood. In this study, we analyzed longitudinal changes in microbial and fungal communities in dry-aged beef using a metagenomic platform. 16S rRNA sequencing revealed that dry aging led to an increase in bacterial diversity, and Actinobacteria and Firmicutes, which are mostly lactic acid bacteria, were dominant on dry-aged beef. However, prolonged dry aging reduced the diversity of lactic acid bacteria. Sequencing of the internal transcribed spacer (ITS) region showed that fungal diversity was reduced by aging and that *Helicostylum* sp. was the most common species. These results suggest that there are various microorganisms on dry-aged beef that interrelate with each other and affect meat quality. Understanding microbial characteristics during the aging process will help to enhance beef quality and functional effects.

## 1. Introduction

In the last two decades, there has been a shift in the consumer lifestyle and eating habits, with a preference for high food quality and premium foods with essential nutrients [1]. With respect to high food quality, there are representative methods for improving the palatability and flavor of beef, including salting, curing, smoking and aging [2,3,4,5]. Among them, beef aging has been of the utmost interest recently for a wider array of purveyors and retail consumers in the United States and Australia and is becoming more popular in Asian countries [6].

Beef aging is a process of storing meat at refrigerated temperatures to enhance tenderness and flavor. In general, there are two forms of beef aging techniques: wet and dry, depending on the degree of dehydration in the meat [6]. Wet aging is a technique for aging meats in a vacuum-sealed bag to retain moisture, while dry-aged beef is unpackaged and left to age for several weeks. Compared to wet aging, dry aging creates a greater concentration of flavor and forms an external crust with certain microbial species on the meat surface [6]. This process provides the unique flavor of dry-aged beef and maximizes palatability [7].

Dry aging involves the growth of various microorganisms on the beef surface, affecting beef quality through their proteolytic and lipolytic activities as well as their metabolic products [8,9]. Although limited studies are available on the effect of microorganisms on dry aging, a few bacteria and yeasts/molds have been reported to be present on dry-aged beef. Yeasts and molds, including *Thamnidium* sp., *Pilaira anomala* and *Debaryomyces hansenii*, are frequently detected in dry-aged beef, which directly affects beef quality by releasing proteases, breaking down myofibrils with collagenolytic enzymes, and producing flavor compounds [5,10]. In contrast to fungal composition, most bacterial analyses have focused on the reduction of pathogenic bacteria, such as generic *Escherichia coli*, coliforms, *E. coli* O157:H7, *Listeria monocytogenes* and *Salmonella* sp., during the process of dry aging [11,12,13]. Recently, we reported that lactic acid bacteria were significantly increased during all dry aging periods, and culturomic analysis at the fungal level showed the presence of *Penicillium camemberti* and *D. hansenii* [14]. However, despite the current findings, there is still a lack of comprehensive understanding and analysis of the microbial effects on dry aging. In particular, to date, there is very little information on the bacterial and fungal diversity, characteristics, and safety of dry-aged beef.

In recent decades, culture-independent methods based on metagenomics analysis have been applied to elucidate the genomic characterization of the microbiome associated with food and meat sciences [5,15,16]. In this study, we aimed to identify the diversity and characteristics of microorganisms on the surface of dry-aged beef based on a metagenomics platform. We compared microbial and mycobial compositions using 16S- and internal transcribed spacer (ITS)-based amplicon sequencing in dry-aged beef at different aging periods. Our results suggested that a number of microbial and fungal communities were present on dry-aged beef, interrelating with each other and affecting the aging process.

## 2. Materials and Methods

### 2.1. Dry Aging of Beef and Sample Collection

Eight carcasses (1st grade Hanwoo cattle) were selected and aged at 1–4 °C and a relative humidity of 80–90% until 160 days after slaughter. At 12-, 30-, 70- and 160-days postmortem during aging, a single 5.0-cm-thick *longissimus thoracis* and *biceps femoris* section was taken from the surface of each carcass. Samples from the dry-aged beef were transported to the laboratory at 4 °C within 3 h after being cut, without being vacuum packed.

### 2.2. DNA Preparation and Sequencing

Ten grams of surface samples were homogenized in 90 mL of Ringer’s solution (Oxoid, Basingstoke, UK), and 1 mL aliquot of the homogenate was centrifuged at 10,000× *g* for 5 min. The pellet containing microorganisms was collected, followed by DNA extraction using the Powerfood Microbial DNA Isolation kit (Mo Bio Laboratories, Inc., Carlsbad, CA, USA) according to the manufacturer’s instructions. Each DNA sample was adjusted to a concentration of 1 ng/µL and subjected to PCR according to the 16S Metagenomic Sequencing Library protocols (Illumina, San Diego, CA, USA). The V4 region of the 16S rRNA genes (primer set: forward, 5′-CCT ACG GGN GGC WGC AG-3′; reverse, 5′-GAC TAC HVG GGT ATC TAA TCC-3′) and the ITS region (primer set: forward, 5′-TCG TCG GCA GCG TCA GAT GTG TAT AAG AGA CAG GCA TCG ATG AAG AAC GCA GC-3′; reverse, 5′-GTC TCG TGG GCT CGG AGA TGT GTA TAA GAG ACA GTC CTC CGC TTA TTG ATA TGC-3′) were analyzed using the Illumina MiSeq platform (Illumina, San Diego, CA, USA). After measuring the concentration of the index PCR products using PicoGreen (Invitrogen, Carlsbad, CA, USA), equimolar PCR amplicons were pooled and sequenced using the MiSeq^®^Reagent Kit v3 (600 cycles) for 301 paired-end bases, following the manufacturer’s protocol based on the MiSeq system platform (Macrogen, Seoul, South Korea). The sequencing result was received in the format of a fastq file.

### 2.3. Metagenomic Analysis

Fastq files obtained from MiSeq paired-end sequencing data were analyzed using the Mothur (v. 1.41). In Mothur, reads were merged using the make.contig command and quality-filtered by the screen.seqs command. We aligned the sequences to the SILVA database v. 138 and the chimeric sequences were removed using the VSEARCH program v2.11.1 [17]. Taxonomic classification was analyzed using the Greengenes-formatted database 14 released in 2013 and then Chloroplast, Archaea, Mitochondria, and Eukaryota sequences were removed from the dataset. Low abundance operational taxonomical units and singletons were removed using the Mothur subroutine “split.abund”, and operational taxonomic units (OTUs) were classified using the distance 0.03 calculation (97% sequence similarity). Rarefaction curves were calculated using Mothur and the OTU and taxonomy table from Mothur were further analyzed on the R platform v. 3.6.2 using the Phyloseq and Vegan packages. Plots were generated using GraphPad Prism 8.0 (San Diego, CA, USA).

### 2.4. Statistical Analysis

All statistical tests were performed using R software (http://www.r-project.org/) to evaluate diversity. The α-diversity indices (Shannon index, Chao1, and Simpson index) were calculated using the vegan package. Taxonomic profiles were used to conduct principal component analysis (PCA) which was performed on log-transformed data using the ADE4 package to analyze the distance matrices for visualization and they plotted against each other to compile the microbiota compositional differences between samples.

## 3. Results

### 3.1. Dry Aging Leads to Changes in Species Diversity in Beef

The dry aging process involves the growth of various microorganisms on the beef surface with interactions between species. To compare compositional changes of microorganisms with meat ripening in dry-aged beef, we conducted next-generation sequencing of bacterial 16S rRNA and ITS sequencing. We selected incubation periods of 12, 30, 70 and 160 days, as numerous researchers have reported the most frequent range for dry-aged beef as between 14 and 40 days [6]. Initially, a total of 1,567,714 and 1,147,534 reads were obtained for 16S and ITS sequences, respectively. The mean number of effective reads per sample was 368,560 and 265,564 for 16S and ITS sequences, respectively, after removing adapter sequences. The ratio of reads that had a phred quality score over 30 (Q30%) ranged from 62.2% to 68.6%. The total assembled sequencing of 16S and ITS sequences came in 874,298 and 875,878, respectively. The filtered read counts of clustering with 97% similarity were 172,274 and 645,514 for 16S and ITS sequences, respectively.

**Figure 1:**
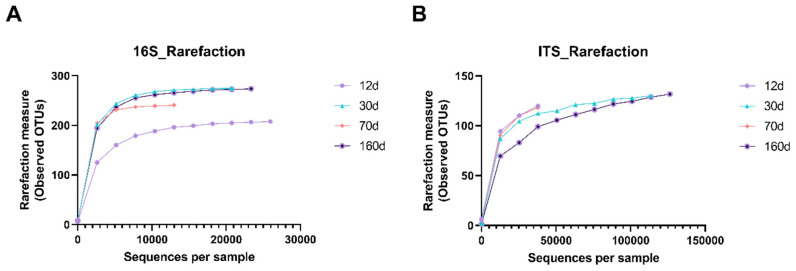
Rarefaction plots of the microbial (**A**) and mycobial (**B**) communities on the surface of dry-aged beef with respect to the aging periods. Rarefaction curves display the number of operational taxonomic units (OTUs) detected based on the sampling intensity of the libraries. ●, day 12; ▲, day 30; ♦, day 70; and ●, day 160.

**Figure 2:**
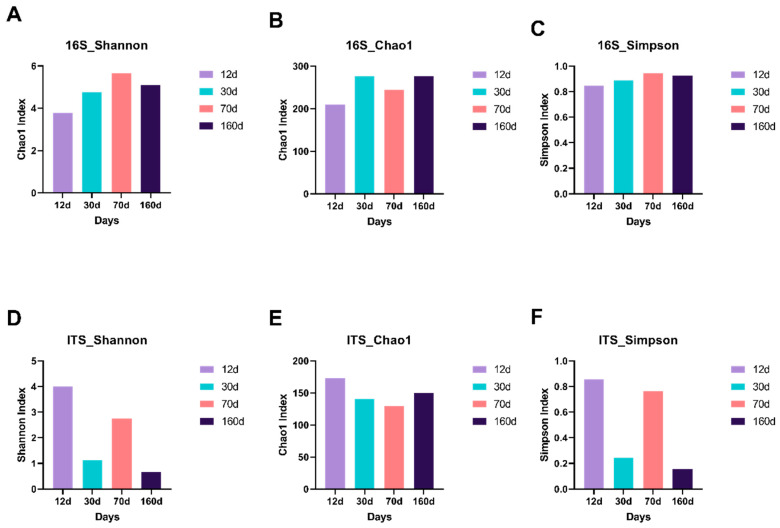
Alpha-diversity indices of the microbial (**A**–**C**) and mycobial (**D**–**F**) communities on the surface of dry-aged beef with respect to the aging periods. Alpha-diversity indices are composite indices reflecting abundance and consistency measured on the basis of Shannon, Chao1, and Simpson indices.

Species richness represents the number of different species in a sample. The rarefaction measure of bacterial 16S rRNA sequencing was lowest at day 12 and increased during the process of beef aging (Figure 1). However, the fungal mycobiome profile did not show a significant trend with time of aging. We next evaluated species diversity, represented by Chao1, Shannon, and Simpson indices, which show how evenly the microbes are distributed in a sample. As shown in Figure 2, all three bacterial species diversity estimates were lowest at day 12 and increased as the aging process continued. In contrast, fungal species diversity indices indicated notably high fungal diversity at day 12, but relevant changes by time were not found. There was a large difference among the fungal species richness estimators between Shannon/Simpson and Chao1, possibly because the Chao richness estimator gives more weight to the low-abundance species, skewing the data sets toward the low-abundance species [18]. These results suggest that dry aging led to bacterial species diversity, while fungal species were influenced differentially during the process.

**Figure 3:**
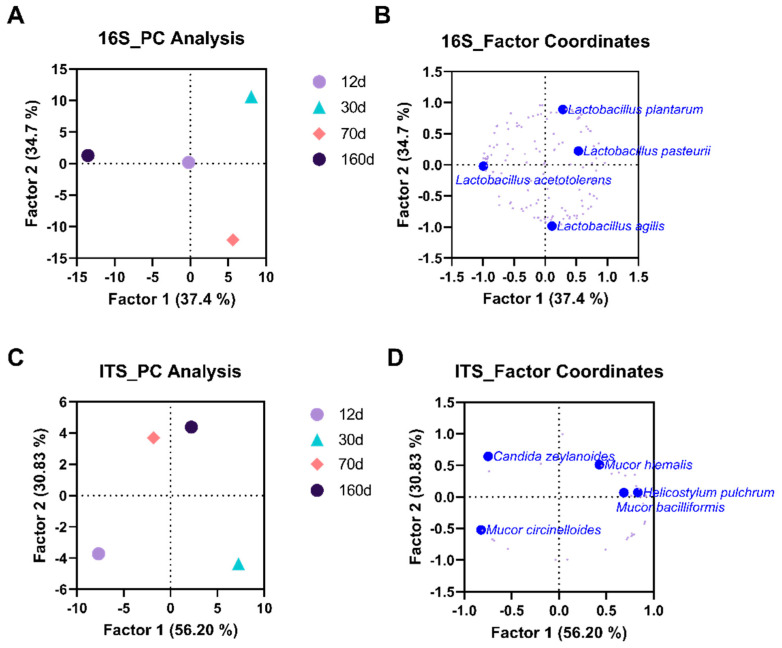
Plots of principal component and classification analysis based on cases (**A**,**C**) and variables (**B**,**D**). Taxonomy abundance counts of the microbial (**A**,**B**) and mycobial (**C**,**D**) communities on the surface of dry-aged beef with respect to the aging periods were projected into principal components based on the correlation matrix. Notable bacterial species on the factor coordinates are indicated (**B**,**D**). ●, day 12; ▲, day 30; ♦, day 70; and ●, day 160.

Principal component and classification analysis were conducted to compute the principal components based on the correlation matrix and visualize the classification of variables and cases (Figure 3). We compared the datasets and confirmed that each group was composed of a distinct microbial species. The fungal communities of dry-aged beef at day 70 and day 160 were more similar than those at the earlier stages of aging. Various species of *Lactobacillus* and *Mucoraceae* family strains were major determinants of the classification for the microbiome and fungal mycobiome, respectively. Overall, metagenomic analysis results showed a complex interrelation of the microbiome and mycobiome with respect to the dry aging process.

### 3.2. Dry Aging Alters Microbial Compositions in Beef

In our previous study, we reported that lactic acid bacteria were significantly increased for 50 days during all dry aging periods [14]. However, there is no information on the individual strain composition of microorganisms in dry-aged beef after 60 days of incubation. Here, we demonstrated the taxonomic composition of the microbiome and mycobiome at the phylum and genus levels by dry aging periods.

**Figure 4:**
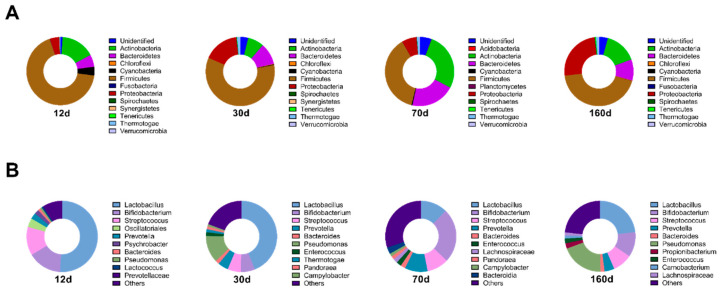
Relative abundance (%) plots of the microbial communities on the surface of dry-aged beef with respect to the aging periods. The top ten most abundant bacteria at the phylum level (**A**) and genus level (**B**) are represented, and the rest of the bacteria were pooled in the ‘others’ category.

At the phylum level, Firmicutes were most dominant in all time periods (Figure 4). The relative abundances of the following two phyla, Actinobacteria and Bacteroidetes, were high but dependent on each period, and dry-aged beef at day 70 had higher Actinobacteria and Bacteroidetes than other time periods. It is noted that the abundance of Cyanobacteria was high only at day 12.

At the genus level, well-known lactic acid bacteria, including *Lactobacillus, Bifidobacterium*, and *Streptococcus*, were the most abundant bacterial strains. At days 12 and 30, the lactic acid bacteria composition was more than 50% of the total detected bacterial abundance, but it decreased with the ripening process. *Pseudomonas* sp., especially *Pseudomonas psychrophila*, was present at high levels at day 30 and day 160 and is considered a pathogenic bacterium causing the deterioration of beef and failure of dry aging. *Prevotella*, known to mainly inhabit the human gut, was present at lower levels on day 12 but increased during aging. The existence of food-borne pathogens, including *Bacillus cereus, Staphylococcus aureus, Listeria monocytogenes*, or *Escherichia coli*, was not detected. From these results, we speculate that Firmicutes, including lactic acid bacteria, are dominant at the beginning of dry aging but reduced by time interacting with other bacterial species.

### 3.3. Dry Aging Alters Fungal Compositions in Beef

**Figure 5:**
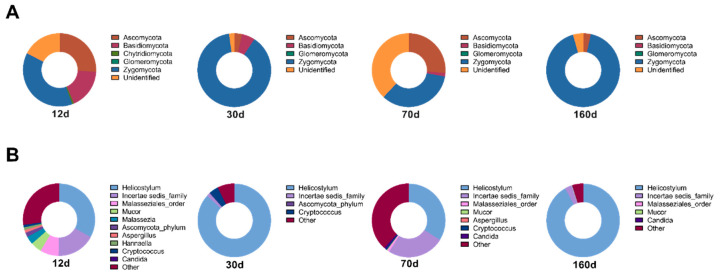
Relative abundance (%) plots of the mycobial communities on the surface of dry aged beef with respect to the aging periods. The top ten most abundant fungi at the phylum level (**A**) and genus level (**B**) are represented, and the rest of the fungal strains were pooled in the ‘others’ category.

The dry aging process encourages the growth of beneficial molds [6]. *Thamnidium* sp. and *D. hansenii* are common microorganisms found in dry-aged beef, while potentially harmful yeasts and molds such as *Candida* sp., *Cladosporium* sp., and *Rhodotorula* sp. are sometimes detected [5]. In the current study, fungal mycobial community analysis showed that Ascomycota and Zygomycota phyla were the dominant taxa in dry-aged beef (Figure 5). In particular, Zygomycota was prevalent at day 30 and day 160. The relative abundance of the Basidiomycota phylum decreased with aging time. Combined with the taxonomic fungal composition at the genus level, most of the Zygomycota phylum was composed of *Helicostylum* sp. (33, 86, 33 and 91% at day 12, 30, 70 and 160, respectively), which is a genus in the family *Mucoraceae*, but its biological activity is unknown. On day 12, *Mucor* sp. and *Malassezia* sp. were present, but their composition decreased as aging progressed. *Cryptococcus* sp., which is an invasive fungus causing cryptococcosis and mostly found in soil, was detected only at day 50. Collectively, fungal communities on dry-aged beef were dominated by Zygomycota, specifically *Helicostylum* sp., and their compositional changes were differentially influenced by the aging process compared to the microbial communities.

### 3.4. Prolonged Dry Aging Reduces the Composition of Lactic acid Bacteria

Lactic acid bacteria function in the preservation of the beef product through the generation of lactic acid during metabolic changes and competition with pathogenic microorganisms [19]. To investigate alteration of the lactic acid bacterial composition in dry-aged beef during maturation, we compared the relative abundance of total lactic acid bacterial strains consisting of four families, *Bifidobacteriaceae, Lactobacillaceae, Leuconostocaceae*, and *Streptococcaceae*.

**Figure 6:**
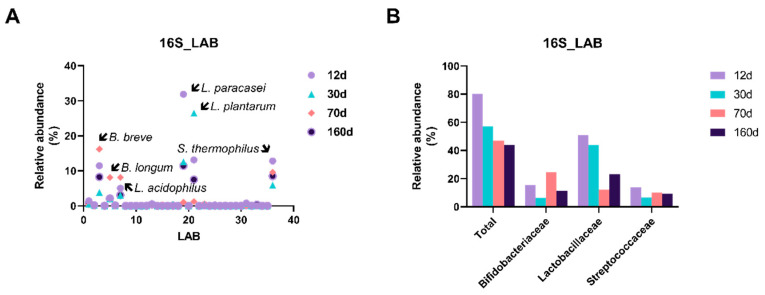
Relative abundance (%) plots of lactic acid bacteria on the surface of dry-aged beef with respect to the aging periods. A total of 36 lactic acid bacterial species (**A**) were detected, including families of *Bifidobacteriaceae, Lactobacillaceae*, and *Streptococcaceae* (**B**). Major species with high relative abundance are indicated in (**A**).

**Figure 7:**
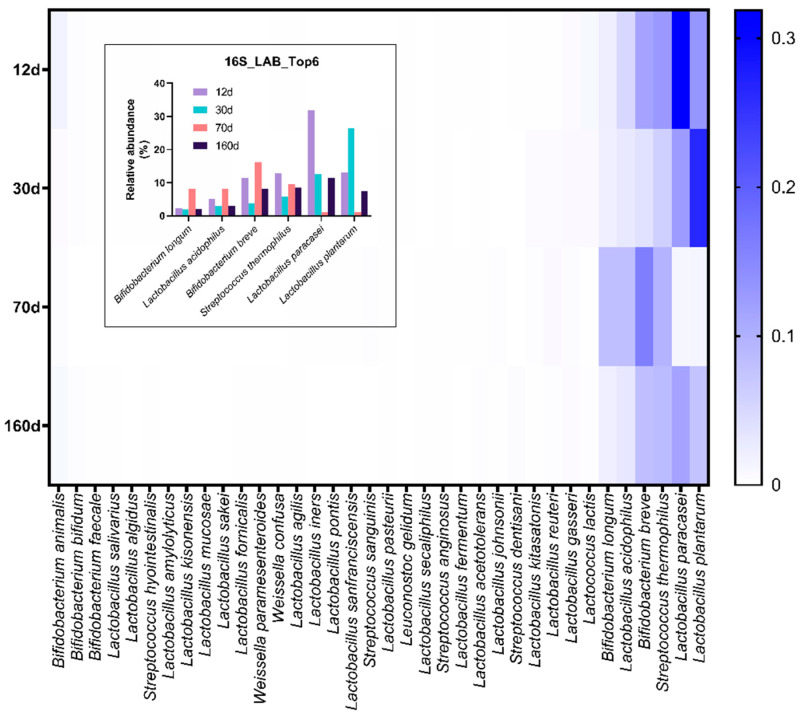
Heatmap of lactic acid bacterial abundance on the surface of dry-aged beef with respect to the aging periods. The blue–white color system was used to represent the relative abundance of each bacterial strain belonging to the families *Bifidobacteriaceae, Lactobacillaceae*, and *Streptococcaceae*. Inset: relative abundance (%) of the most dominant lactic acid bacterial species on dry-aged beef.

At day 12, the bacterial family composition occupied 80.16% of the total bacterial species but gradually decreased to 43.78% at day 160. Each bacterial family composition was different among the aging periods. For example, *Lactobacillaceae* was the most prevalent family at day 12 and day 30, while *Bifidobacteriaceae* was dominant at day 70. As shown in Figure 6 and Figure 7, *Bifidobacterium breve* and *Bifidobacterium longum* were more abundant in the day 70 sample, but *Lactobacillus paracasei* and *Lactobacillus plantarum* were more abundant on days 12 and 30. This result implies that relatively aerobic *Lactobacillaceae* would exist during the earlier period of dry aging and then alternate with anaerobic bifidobacterial strains as oxygen availability decreased.

## 4. Discussion

Dry aging is a process of storing meat at refrigerated temperatures, and various microorganisms are involved in enhancing the meat aging process. In this study, we compared microbial and mycobial compositions based on a metagenomics platform in dry-aged beef at different aging periods. Bacterial species diversity increased during the process, while fungal diversity was highest at the beginning of aging. At the phylum level, Actinobacteria and Firmicutes, mostly lactic acid bacteria, were dominant but were reduced in relative abundance by aging. Proteobacteria, mostly *Pseudomonas*, were detected at day 30 and day 160. The dry aging process also altered fungal composition with *Helicostylum* sp. as the most dominant strain, while the relative abundances of *Mucor* sp. and *Malassezia* sp. decreased during the aging process.

In the current study, we showed a high relative abundance of lactic acid bacteria on dry-aged beef until 30 days of aging, but their composition decreased as aging proceeded. To date, only a few studies have been associated with lactic acid bacteria in dry-aged beef. Oh et al. reported increased lactic acid bacterial composition from approximately log 2 colony forming units (CFU) to log 3 CFU in 7 days of wrap packaging after completion of aging [5]. In the study by Ryu et al., lactic acid bacterial abundance increased to log 6 CFU on the surface of dry-aged beef samples of *longissimus thoracis* and *biceps femoris* during aging for 60 days [14]. Lactic acid bacteria are frequently applied as starter cultures in the production of salami, yielding a sour meat aroma and oily mouth feel as well as inhibiting pathogenic bacterial growth by bacteriocins [20,21,22]. Considering that the usual dry aging period is less than 30 days, lactic acid bacteria would be dominant, conferring beneficial effects on dry-aged beef.

Meat deterioration by pathogenic bacteria is problematic. In the current study, we detected *Pseudomonas* sp., mostly *Pseudomonas psychrophila.* The strain is a cold-adaptable facultatively psychrophilic bacterium and can grow at −1 to 35 °C with maximum growth at 25 °C [23,24]. It is a major spoilage organism found in fish meats or food processing facilities [25,26]. Infection by *P. psychrophila* could result in the degradation of proteins and the production of volatile nitrogen-based metabolites such as putrescine and ammonia [27]. *P. psychrophila* has never been reported in livestock, but it is possible for the strain to be present on dry-aged beef during the process of packaging or storage. Notably, the bacterial strain appeared at day 30, but its presence was reduced at day 70, possibly by the interaction with total microbiota in the beef sample, and then appeared again at day 160. To reduce potential infection by the organism, caution is required regarding processing management practices.

Recently, Capouya et al. reported a survey of microbial communities on dry-aged beef processed from commercial dry aging facilities [28]. They found that the microbial community structures were highly dependent on the processing facilities and may be associated with beneficial enzymatic digestion of the tissue contributing to beef quality. Although their specific microbial and mycobial taxa and distributions were different from our current study, *Pseudomonas* sp. and *Mucor* sp. were found to be present in both studies, establishing a general core microbiome for dry-aged beef. It is noted that *Lactobacillius* genus was also found in their study to have a relative abundance of 36.57% in a facility but less than 1% abundance in other locations.

In general, various yeasts and molds can grow on the surface of dry-aged beef, including *Thamnidium* sp., *Pilaira anomala* and *D. hansenii* [5,14]. In this study, we first report the presence of *Helicostylum* sp. based on the metagenomic platform. It was described by Corda in 1842 and has a sporangium without an apophysis, forming globose sporangiola borne on straight or recurved branches and lacking stolons and rhizoids [29]. However, its biological and physiological characteristics are still unidentified. Interestingly, the order of *Malasseziales*, including *Malassezia* sp., was only detected at the beginning of dry aging. *Malassezia* is naturally found on the skin surfaces of many animals, sometimes causing hypo- or hyper-pigmentation [30]. In a recent paper, the presence of *Malassezia* was reported in dried crimson snapper during storage for 50 days [31]. It is speculated that the bacteria could be included from cows or during the preparation process by purveyors, and their composition could be reduced by interacting with microbial communities as aging proceeds. However, a possible route of contamination needs a more specific investigation.

## 5. Conclusions

In the present study, we determined the characteristics of microorganisms on the surface of dry-aged beef based on the metagenomic analysis. We found the longitudinal changes of microbial and mycobial compositions, including lactic acid bacteria. Overall, the current study clearly shows that there are various microorganisms on dry-aged beef interrelating with each other and affecting the meat aging process. Understanding microbial characteristics during aging will help to enhance beef quality and functional effects.
